# Supplementary material for: Dendritic NMDA spikes are necessary for timing-dependent associative LTP in CA3 pyramidal cells
Source: Nat Commun. 2016 Nov 16;7:13480. doi: 10.1038/ncomms13480 (PMC5116082; doi:10.1038/ncomms13480)
Supplement: Supplementary Information — Supplementary Figures 1-11 [file ncomms13480-s1.pdf]

**SUPPLEMENTARY INFORMATION**

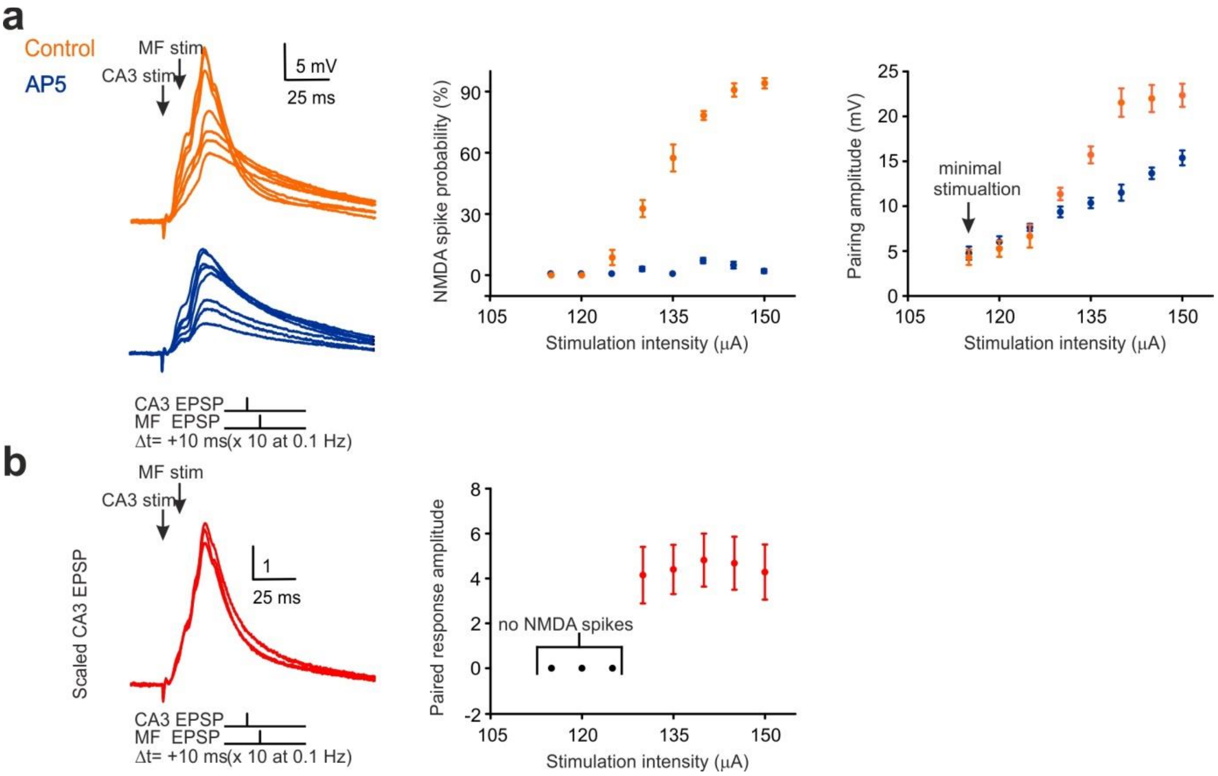


**Supplementary Figure 1**

**The supralinear events evoked in CA3 pyramidal cells fulfill the criteria for NMDA spikes, exhibiting a threshold, sensitivity to NMDAR blockade, and all-or-none expression.**

**(a)** Left panel: Experimental design: each trace is an average of 10 sweeps obtained by pairing a rCA3 EPSP followed by a MF EPSP according to the ITDP protocol shown below. Families of traces were generated by progressively increasing the stimulation intensity beyond threshold intensity under control conditions and after NMDAR blockade. Center panel: Pooled data for the probability of triggering a supralinear response as a function of stimulation intensity ($n$ = 5). As NMDA spikes are all-or-none events[17], the response amplitude is expected to increase abruptly once that threshold is exceeded. However, as the data points represent responses averaged over ten trials that include both failures and successes, the amplitude increases incrementally reflecting the change in probability of surpassing threshold with stronger stimulation. Right panel: averaged responses showing the change in amplitude of the paired EPSP with incrementing stimulation intensity. In the presence of D-AP5 supralinear events are prevented and the amplitude of the averaged responses with increasing stimulation intensity follows a linear function mediated by AMPA receptors. **(b)** The all-or-none nature of the NMDA spike is revealed when failures are excluded and the amplitude of only supralinear events is plotted, which is consistent with the bimodal distribution of rCA3/MF summated responses (**Fig. 1c**; Brandalise and Gerber, 2014). It should be noted that although NMDA spikes are all-or-none events, their amplitude

is determined by the number of synapses activated and can therefore vary from trial to trial if the number of stimulated inputs changes. However, when data from several cells are averaged, this variability is no longer apparent.

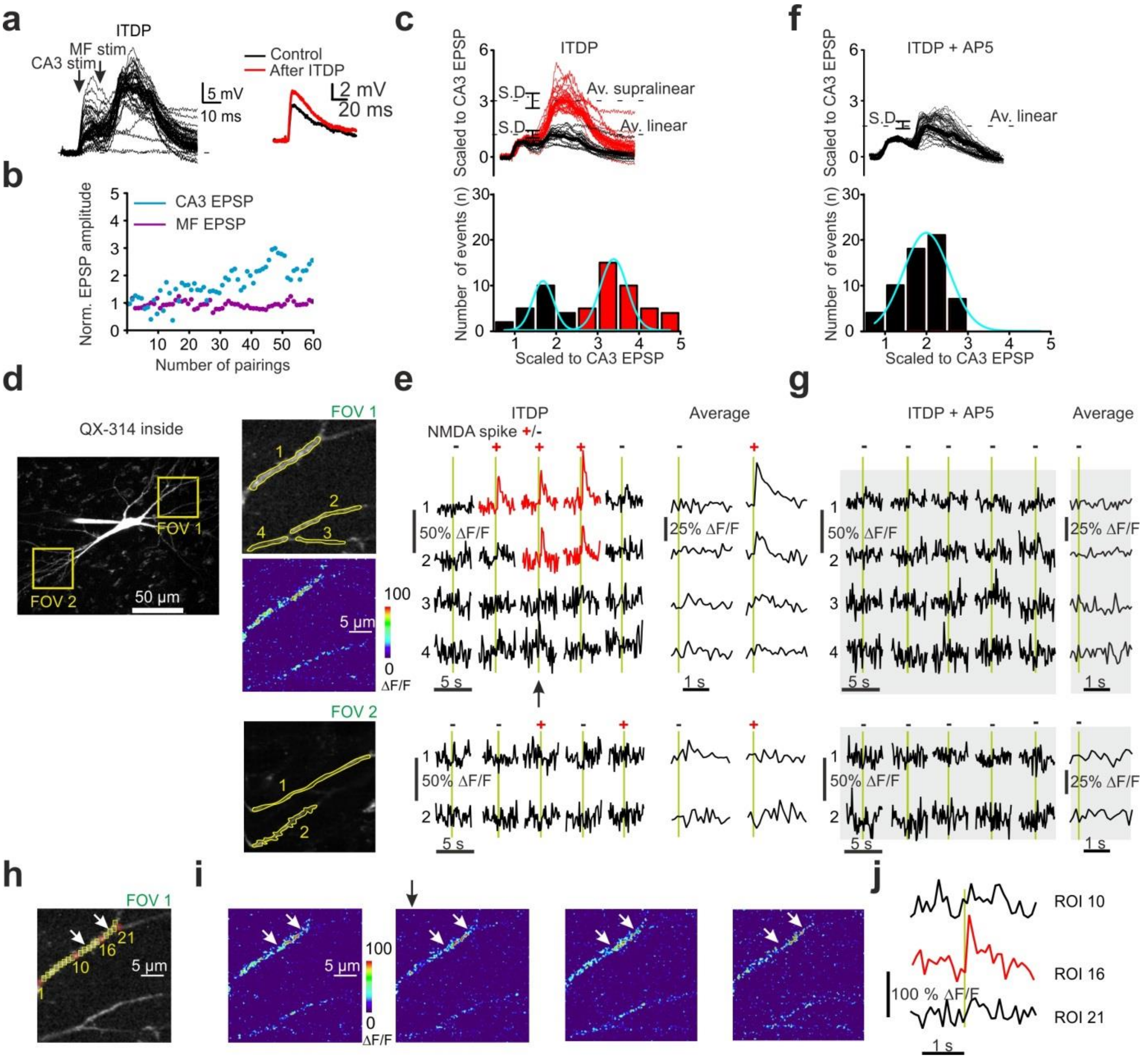


**Supplementary Figure 2**

**Peak dendritic $Ca^{2+}$ transients associated with NMDA spikes exhibit highly consistent inter-trial localization.**

**(a)** Raw data recorded during the ITDP protocol from a representative CA3 pyramidal cell in which sodium currents were blocked with intracellular QX-314 (500 µM) to prevent back-propagating APs and dendritic sodium spikes. After 60 pairings, the rCA3 EPSP undergoes LTP. **(b)** Time course of the experiment showing that rCA3-evoked EPSPs were potentiated whereas MF-evoked EPSPs were not at the ITDP stimulation frequency of 0.1 Hz. **(c)** The same 60 traces were scaled to normalize the amplitude of the evoked rCA3 EPSP that precedes the MF EPSP. This procedure reveals the presence of linear (black) as well as supralinear events (red), which are bimodally distributed. Note that at the end of the 60 pairings the rCA3 EPSP is potentiated by 84.9 ± 10.6% as a consequence of short-term facilitation. **(d)** Responses were analyzed in the basal (FOV 1) and apical dendrites (FOV 2). In FOV 1, two of four dendritic segments exhibit $Ca^{2+}$ transients during NMDA spikes. The same stimulation failed to evoke $Ca^{2+}$ transients in the dendrites imaged in FOV 2. **(e)** Dendritic $Ca^{2+}$ transients evoked during 5 consecutive pairings (green bars) of rCA3 and MF

EPSPs in which “+” and “-” mark the trials associated with supralinear and linear EPSPs, respectively. Averaged $Ca^{2+}$ transients are from 30 consecutive pairings. The arrow indicates the trial corresponding to the image showing the change in Fluo-5F fluorescence in d. Averaged responses are shown on the right. **(f)** NMDA spikes are no longer observed after blocking NMDARs (D-AP5) and, consequently, the amplitudes of the events exhibit a unimodal distribution. **(g)** When NMDA spikes are prevented, dendritic $Ca^{2+}$ transients are not observed. **(h)** A series of uniformly sized ROIs (~1 µm x 1 µm) numbered from 1 – 21 were positioned along a responsive dendritic segment as identified by the heat map in d. **(i)** Images from four consecutive pairing trials in which an NMDA spike was evoked. The $Ca^{2+}$ hotspot lies at the center of the dendritic section demarcated by the white arrows (ROI 16). The black arrow indicates the trial corresponding to the traces in j. **(j)** Time-course of the calcium response associated with an NMDA spike for ROI 16 (red trace) and for ROIs outside the white arrows (black traces). For pooled data of NMDA spike magnitude see Supplementary Fig. 4b.

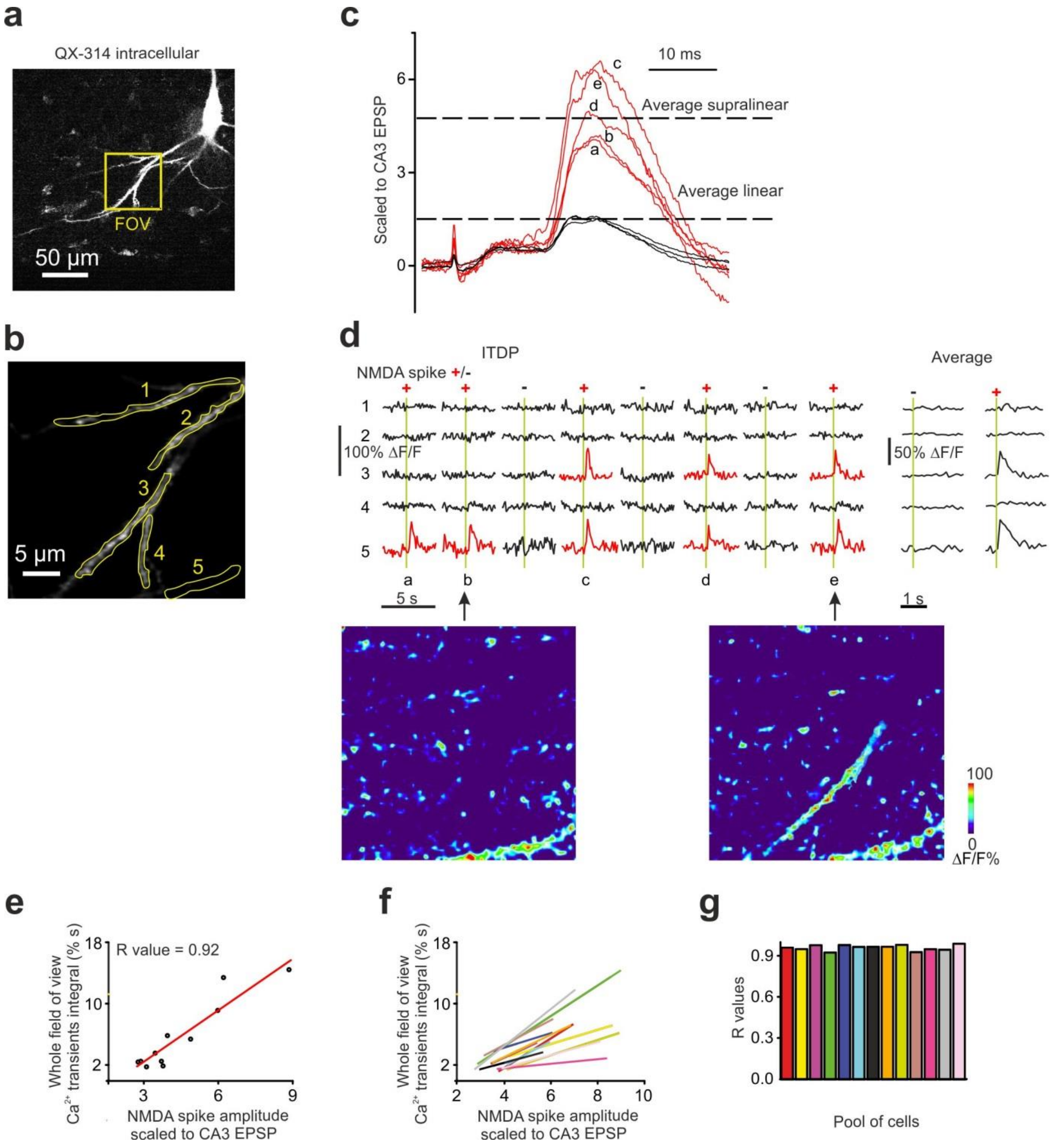


**Supplementary Figure 3**

**Event analysis showing a correlation between the number of branches exhibiting dendritic $Ca^{2+}$ transients and the amplitude of electrophysiologically recorded NMDA spikes.**

**(a)** FOV in the basal dendrites. **(b)** Dendritic $Ca^{2+}$ transients were analyzed in five ROIs. **(c)** Eight consecutive superimposed events evoked by pairing a rCA3 EPSP with a subsequent MF EPSP (normalized to the amplitude of the rCA3 EPSP) which generated three linear responses and five supralinear responses generated by NMDA spikes. **(d)** Fluo-5F fluorescence imaging shows that in most cases the dendritic $Ca^{2+}$ transients in these ROIs are associated with NMDA spikes (+) and are restricted to specific branch segments (for this cell: ROIs 1,3,5,6). The arrow indicates the trial for the image showing the change in Fluo-5F

fluorescence in B. **(e)** Correlation between the spatially and temporally integrated $Ca^{2+}$ transient magnitude (integrated over the whole FOV shown in b; 2-s post-stimulation integration time window; in units of "%s") and the NMDA spike amplitude, normalized to the amplitude of the evoked rCA3 EPSP preceding MF stimulation, for the example cell shown in a-d. Only supralinear events were analyzed. **(f)** Same as in e, plotting linear regression lines for all recorded cells shown in Figure 1i. **(g)** Comparison of correlation coefficients for all regression lines shown in f. Mean correlation coefficient $r = 0.96 \pm 0.01$, $n = 13$.

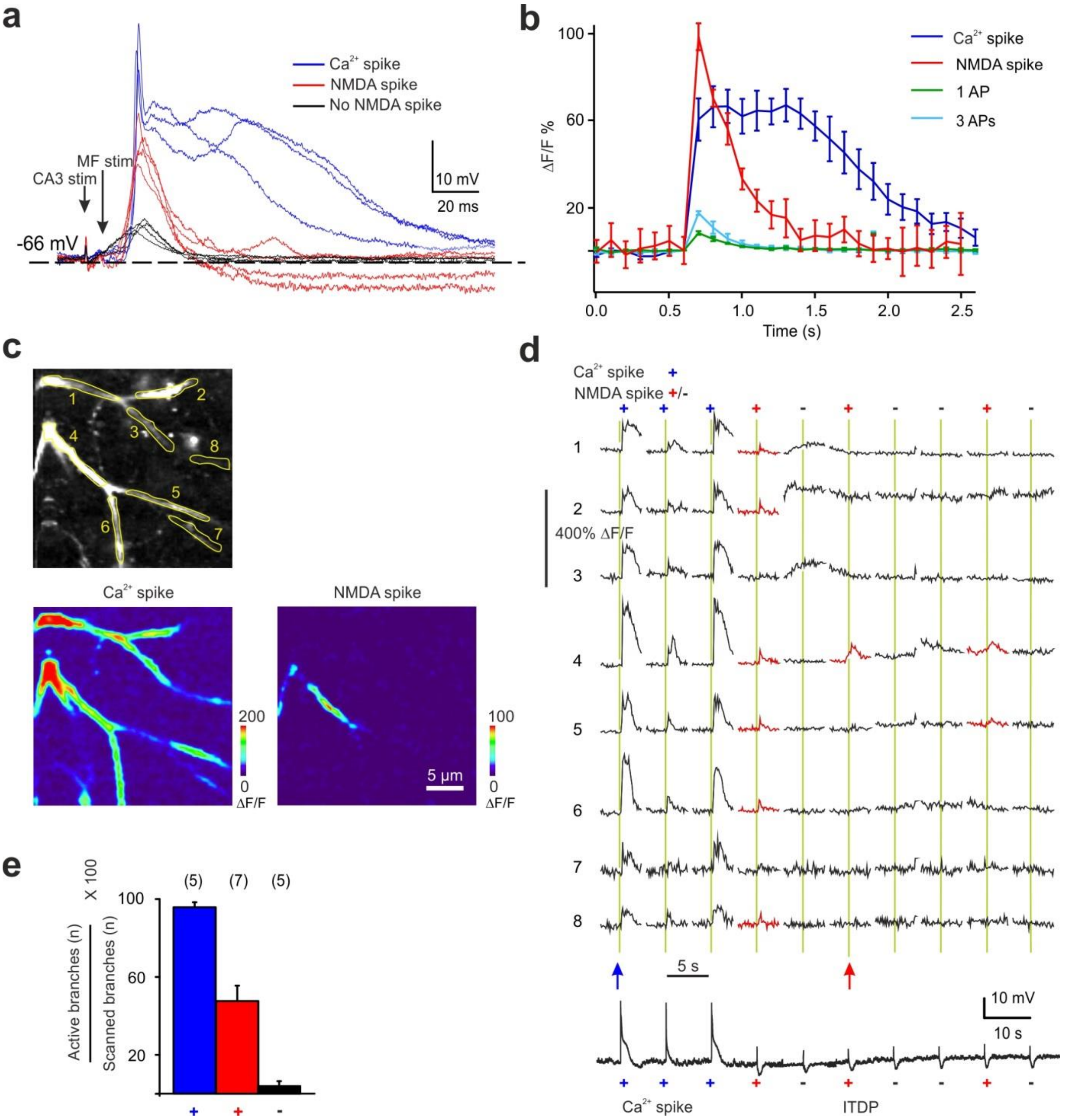


**Supplementary Figure 4**

**Comparison of dendritic $Ca^{2+}$ transients evoked either by NMDA spikes, by voltage-gated $Ca^{2+}$ channels, or by back-propagating APs.**

**(a)** Responses in current clamp mode obtained by sequential pairing of a rCA3 EPSP and a MF EPSP with a delay of 10 ms. Incrementing stimulation intensities evoke first a linear response, then a supralinear NMDA spike, and finally a supralinear $Ca^{2+}$ spike. **(b)** Distinct dendritic $Ca^{2+}$ transients associated with various dendritic electrical responses ($n$ = 5). To allow comparison of response magnitudes, the same spatially restricted ROI (1 µm x 1 µm) was focused on the hotspot generated by the dendritic NMDA spike in the heat map to plot the averaged $Ca^{2+}$ transients corresponding to the different responses (see Fig.1h,1; Supplementary Fig. 2h–j). $Ca^{2+}$ transient integrals over a 2 s window were 121.1 ± 19.9%s ($Ca^{2+}$ spike), 30.4 ± 4.5%s (NMDA spike), 2.0 ± 0.3%s (1AP), and 3.1 ± 1.2%s (3 APs). **(c)** Dendritic ROIs selected for the analysis of $Ca^{2+}$ transients associated with an NMDA spike as

compared with a $Ca^{2+}$ spike. $Ca^{2+}$ spikes are associated with a generalized dendritic $Ca^{2+}$ signal, whereas the $Ca^{2+}$ transients seen with NMDA spikes are spatially restricted. **(d)** Responses to 10 consecutive paired stimuli imaged in 8 ROIs. A blue "+" indicates $Ca^{2+}$ spikes, which were evoked by suprathreshold stimuli, that is, sufficient to evoke APs; a red "+" indicates NMDA spikes, which were generated by subthreshold stimuli; a black "-" indicates trials in which the same intensity subthreshold stimuli elicited linear responses. Note that whereas NMDA spikes trigger $Ca^{2+}$ transients that are restricted to a small subset of dendritic segments, $Ca^{2+}$ spikes lead to a generalized response that is detected in all imaged dendritic branches. The blue arrow indicates traces corresponding to the image labeled $Ca^{2+}$ spike in a. The red arrow indicates traces corresponding to the image labeled NMDA spike in A. Lowermost trace shows the current clamp recording during this imaging experiment. **(e)** Pooled data showing the proportion of imaged dendritic branches within a responsive FOV that exhibited a $Ca^{2+}$ transient during a $Ca^{2+}$ spike (blue) (95.5 ± 2.2%; $n$ = 5) , an NMDA spike (red) (52.6 ± 5.6%, $n$ = 5), or no dendritic spike (black) (4.0 ± 2.2%, $n$ = 5).

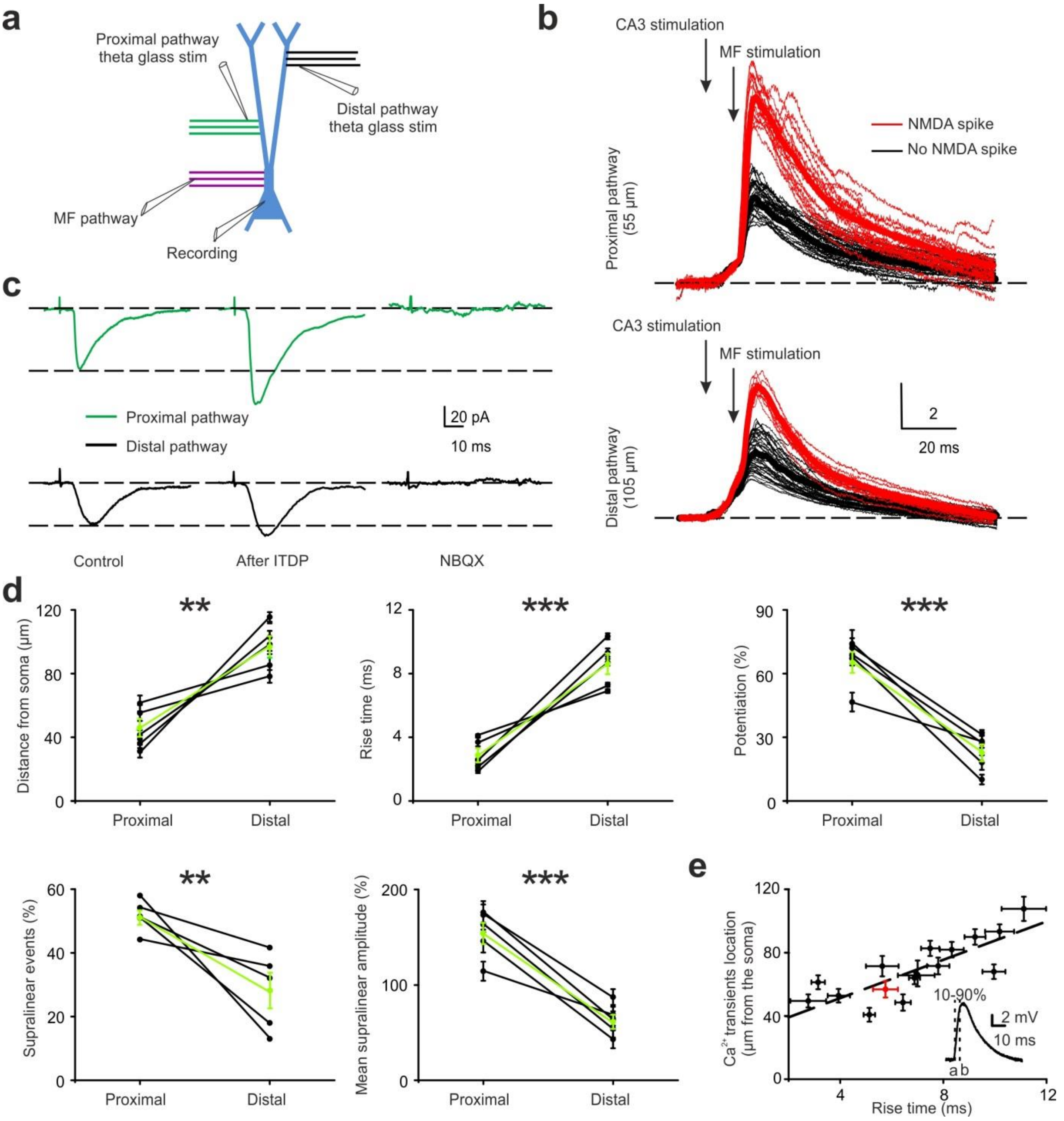


**Supplementary Figure 5**

**Focal dendritic stimulation with theta glass electrodes confirms that the potentiation of rCA3 synapses with the ITDP protocol decreases as a function of their distance from the soma.**

**(a)** Experimental configuration with two theta glass stimulating electrodes positioned close to the dendrites at two different distances from the MF input, which is always close to the soma. **(b)** Responses to 60 repetitive pairings of a rCA3 EPSP followed by a MF EPSP to induce ITDP, normalized to the amplitude of the rCA3 EPSP. Both the amplitude and the probability for generating an NMDA spike are greater at proximal synapses versus distal synapses. **(c)** Averaged traces for rCA3 EPSCs showing a faster rise time and greater LTP at proximal versus distal synapses. **(d)** Pooled data comparing the rise time (proximal: 2.9 ± 0.4 ms, $n$ = 5; distal: 8.6 ± 0.6 ms, $n$ = 5, $P$ = 0.006), the distance from soma of the stimulation electrode (proximal: 45.6 ± 5.6 µm, $n$ = 5; distal: 23.0 ± 3.9 µm, $n$ = 5, $P$ < 0.001), the amount of

potentiation (proximal: 65.6 ± 4.9%, $n = 5$; distal: 96.4 ± 6.8%, $n = 5$, $P < 0.001$), the number of NMDA spikes occurring during the 60 pairings (proximal: 51.7 ± 2.3%, $n = 5$; distal: 28.9 ± 5.6%, $n = 5$, $P < 0.005$), and the mean amplitude of the evoked NMDA spikes (proximal: 156.3 ± 11.1%, $n = 5$; distal: 63.8 ± 7.4%, $n = 5$, $P < 0.001$). **(e)** Pooled data for cells plotted in Fig. 1h demonstrating the positional correspondence of evoked EPSPs and imaged $Ca^{2+}$ transients. rCA3 EPSP rise time (as a proxy for distance of the evoked EPSP from the recording electrode) was plotted against distance from the soma where $Ca^{2+}$ transients were detected ($r = 0.79 \pm 0.01$, $n = 13$). The red data point corresponds to the cell shown in Fig. 1.

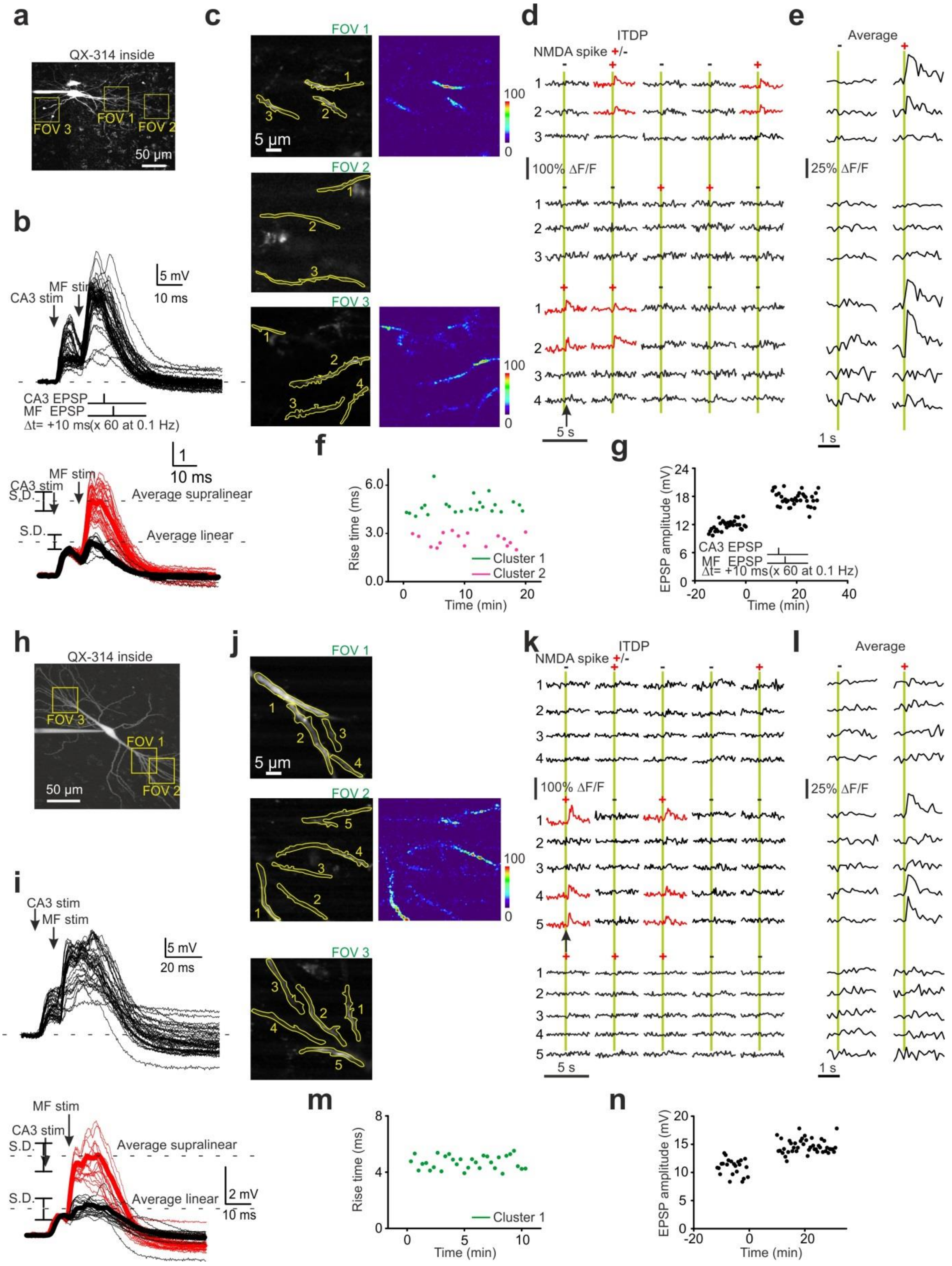


**Supplementary Figure 6**

**In some experiments, stimulation led to activation of two spatially distinct groups of rCA3 inputs to a CA3 pyramidal cell evoking differentially localized dendritic $Ca^{2+}$ transients.**

**(a)** A CA3 pyramidal cell labeled with Fluo-5F (100 µM) and Alexa 495 (10 µM) in which three fields of view were selected for analysis. **(b)** Above are raw data and below are traces normalized to the amplitude of the evoked rCA3 EPSP preceding the MF stimulus during 60 repetitive pairings (bottom). **(c)** ROIs in which dendritic $Ca^{2+}$ transients were analyzed showing that $Ca^{2+}$ transients were evoked in FOV 1 and FOV 2. **(d)** Responses to 5 representative consecutive pairings (green bars) imaged in the 3 fields of view during the ITDP protocol. **(e)** Averaged traces reveal that dendritic $Ca^{2+}$ transients were associated with NMDA spikes (+) and were generated in two distinct circumscribed areas of the dendritic tree (FOV 1 in the apical part of the dendritic tree and FOV 3 in the basal tree). **(f)** Plotting the rCA3 EPSP rise time during the control period preceding the ITDP protocol revealed a bimodal distribution (cluster 1 and cluster 2), consistent with the stimulation of two distinct groups of synapses localized in different parts of the dendritic tree. This phenomenon, in which two separate areas of the dendritic tree were activated with one extracellular stimulation electrode, was observed in 3 out of 13 cells. **(g)** Time course for LTP induction at the rCA3 input in response to the ITDP protocol. **(h-n)** Data from a similar experiment recorded from a different CA3 pyramidal cell. In this case, however, plotting the rCA3 EPSP rise time during the control period preceding the ITDP protocol revealed a homogeneous distribution indicating that the stimulated input activates a single clustered group of synapses. This pattern of dendritic activation fits with the imaging data in which $Ca^{2+}$ transients were observed in only one FOV.

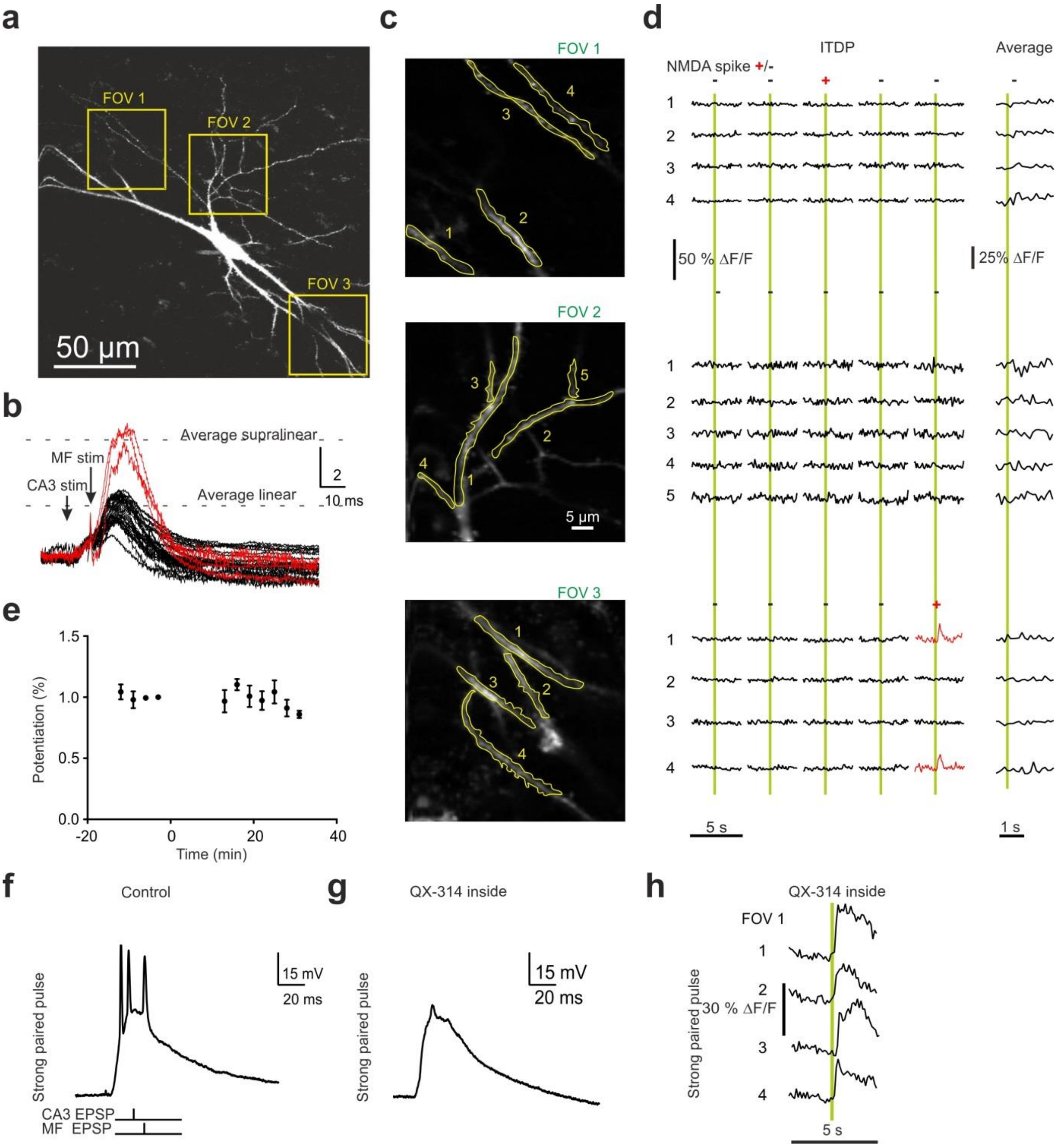


**Supplementary Figure 7**

**When ITDP fails to trigger NMDA spikes, dendritic $Ca^{2+}$ transients are not detected and LTP is not induced.**

**(a)** One of the group of three CA3 pyramidal cells represented as green data points in Figure 1h. **(b)** In these cells an NMDA spike was generated in less than 15% of the 60 pairings during the ITDP protocol. Responses are shown normalized to the amplitude of the rCA3 EPSP. **(c)** Images from three FOVs in regions of the dendritic tree indicated in a. **(d)** Recordings in the three FOVs during 5 consecutive pairings (green bars) of the ITDP protocol. Note that for this cell dendritic $Ca^{2+}$ transients were almost never detected in any of the three FOVs. **(e)** Time course of the mean amplitude of the rCA3 EPSP before and after the ITDP protocol for the three cells represented as green data points in Figure 1h in which NMDA spikes were triggered rarely and LTP was not induced. **(f)** A positive control showing

that the absence of NMDA-spike-mediated $Ca^{2+}$ transients is not due to a failure of Fluo-5F dialysis. At the beginning of the recording, prior to intracellular dialysis with QX-314, a high intensity paired pulse stimulus induces a complex $Ca^{2+}$ spike. **(g)** After QX-314 dialysis the same intensity of stimulation evokes a complex $Ca^{2+}$ spike without spikelets (as described previously[31]). **(h)** $Ca^{2+}$ transients were detected in all ROIs during the occurrence of a complex $Ca^{2+}$ spike.

**a**

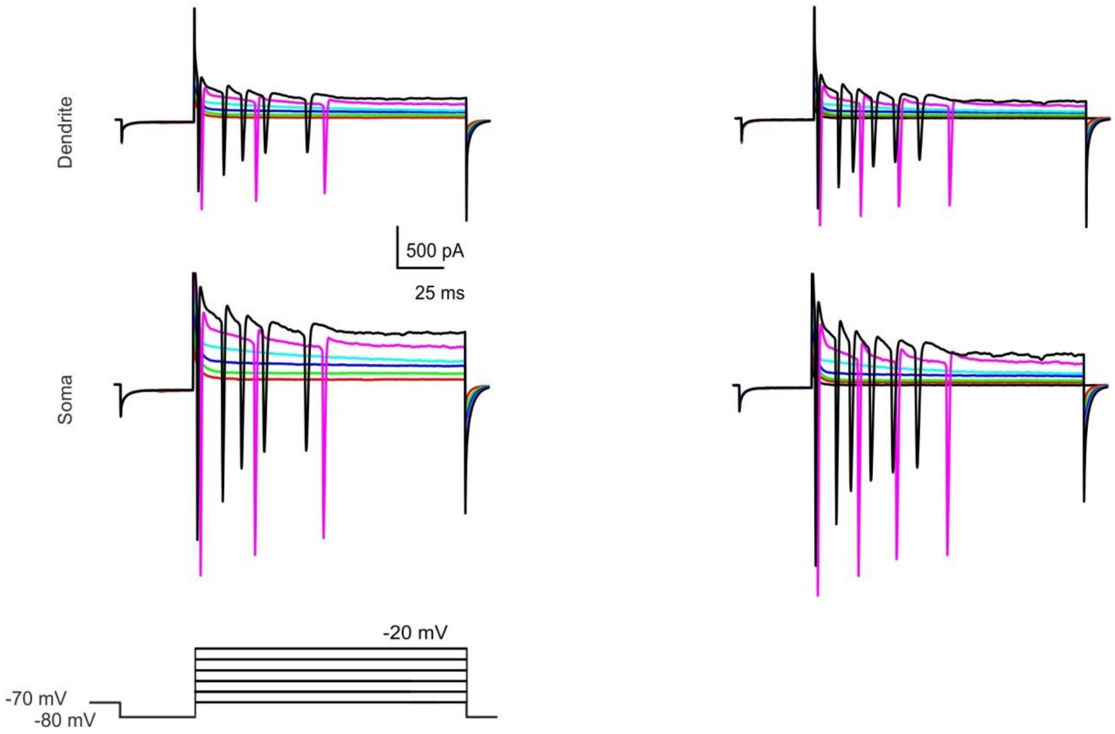


**b**

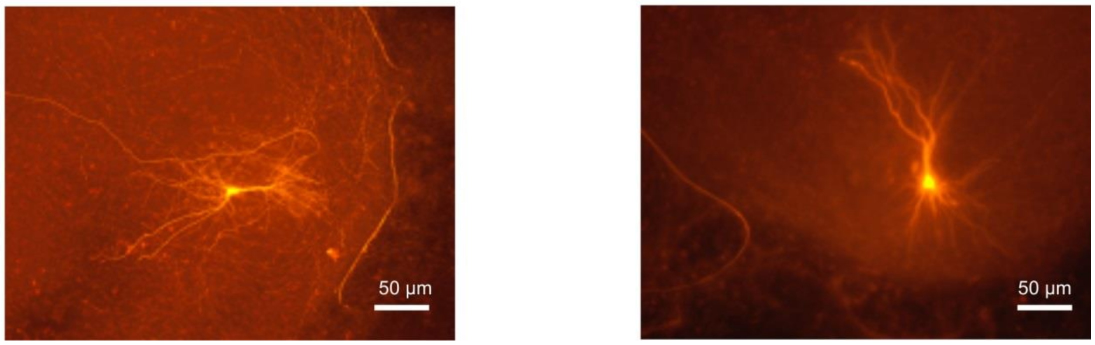


**Supplementary Figure 8**

**Criteria to confirm that the electrodes are targeting the same neuron in dual patch recordings from the soma and a dendrite.**

**(a)** At the beginning of experiments with dual patch recordings from the same cell, voltage steps (inset) were applied to the voltage-clamped CA3 pyramidal cell through the somatic electrode. The synchronicity of the evoked suprathreshold responses (action currents) indicates that both electrodes were contacting the same cell, thereby ruling out synaptic responses between the two cells. **(b)** Biocytin was included in both recording pipettes and slices were fixed in PFA after experiments. Immunostaining revealed only one labeled neuron per slice confirming that both patch pipettes were in the same neuron.

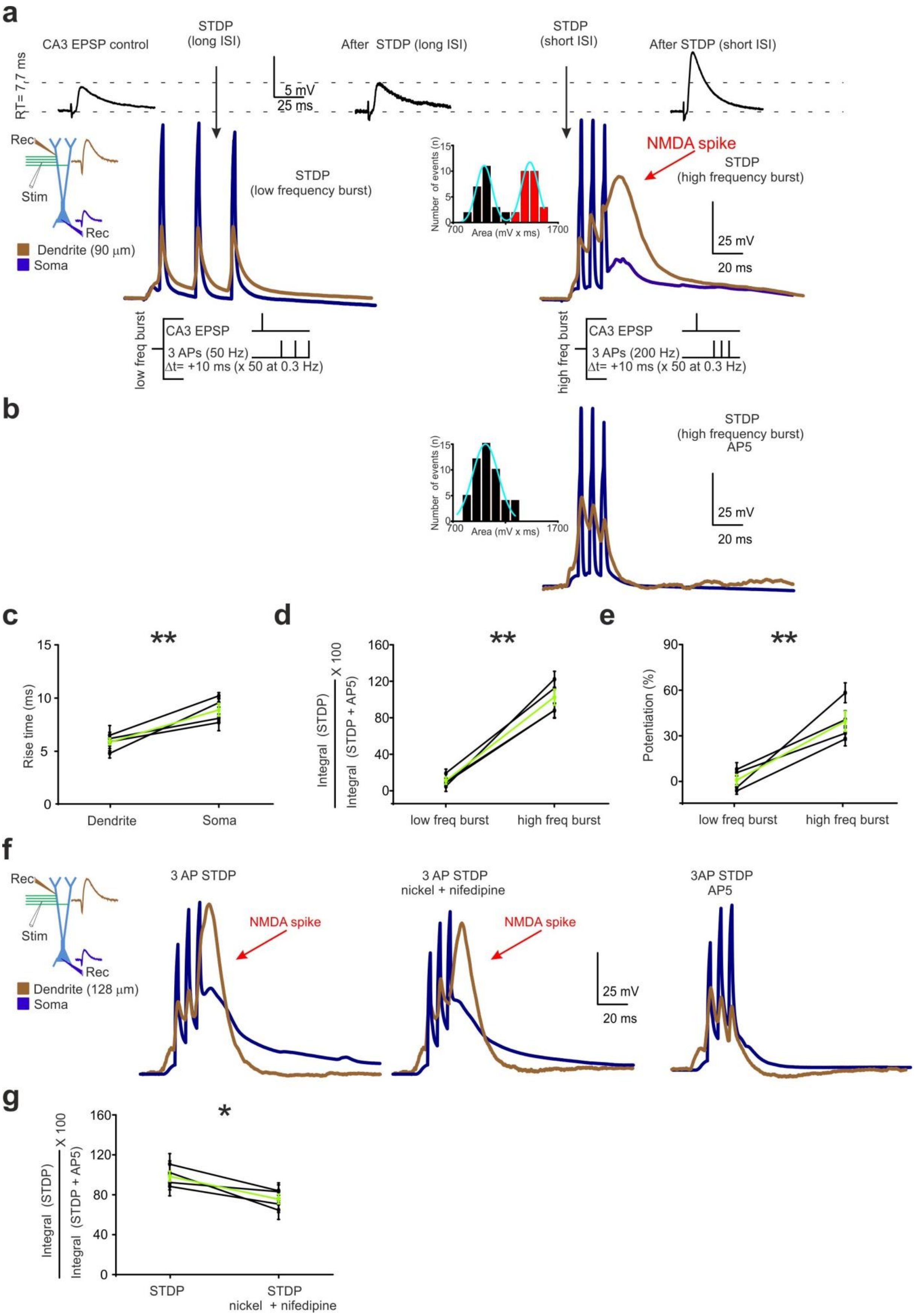

a
CA3 EPSP control
STDP (long ISI)
After STDP (long ISI)
STDP (short ISI)
After STDP (short ISI)
RT= 7.7 ms
5 mV
25 ms
Rec
Stim
Rec
Dendrite (90 µm)
Soma
STDP (low frequency burst)
NMDA spike
STDP (high frequency burst)
Number of events (n)
Area (mV x ms)
25 mV
20 ms
low freq burst
CA3 EPSP
3 APs (50 Hz)
Δt= +10 ms (x 50 at 0.3 Hz)
high freq burst
CA3 EPSP
3 APs (200 Hz)
Δt= +10 ms (x 50 at 0.3 Hz)
b
STDP (high frequency burst) AP5
Number of events (n)
Area (mV x ms)
25 mV
20 ms
c
Rise time (ms)
Dendrite
Soma
d
Integral (STDP) / Integral (STDP + AP5) X 100
low freq burst
high freq burst
e
Potentiation (%)
low freq burst
high freq burst
f
Rec
Stim
Rec
Dendrite (128 µm)
Soma
3 AP STDP
3 AP STDP nickel + nifedipine
3AP STDP AP5
NMDA spike
NMDA spike
25 mV
20 ms
g
Integral (STDP) / Integral (STDP + AP5) X 100
STDP
STDP nickel + nifedipine

**Supplementary Figure 9**

**A critical frequency of AP firing must be exceeded during STDP in order to evoke an NMDA spike and to induce LTP.**

**(a)** Representative traces from experiments in which simultaneous recordings from the soma and from a second order dendrite are compared during a standard STDP protocol consisting of a rCA3 EPSP followed by three APs at low (50 Hz) or high frequency (200 Hz) evoked by a three pulse current injection (2 ms; 4 nA for each AP). rCA3 EPSPs do not potentiate with the low frequency protocol but undergo LTP with the high frequency STDP protocol (black traces: rCA3 EPSPs recorded in the dendrite). Below: Only the high frequency STDP protocol reliably generates a supralinear response (62.8 ± 8.8%, $n$ = 5, $P$ < 0.001). EPSP–AP pairing with a low-frequency (50 Hz) burst of APs failed to trigger NMDA spikes and to induce LTP. Inset: Area under the evoked responses was plotted to distinguish between linear and supralinear events. **(b)** The supralinear response is abolished following NMDAR blockade (D-AP5). Inset: Supralinear responses are prevented when NMDARs are blocked. **(c)** Faster EPSP rise times in dendrite versus soma indicates that for these experiments a majority of the synapses activated by stimulation of rCA3 collaterals were located at or near the dendritic branch recorded from. The green line denotes the pooled average of all cells. **(d)** The contribution mediated by NMDARs to responses generated by the low and high frequency STDP protocols was quantified by calculating the ratio between the areas under the traces in the absence and the presence of D-AP5 (low frequency burst: 10.6 ± 3.0%, high frequency burst: 102.5 ± 8.7%, $n$ = 5, $P$ < 0.001). **(e)** Pooled data showing that potentiation at the rCA3 synapse is only induced in response to the high frequency STDP protocol (low frequency: 2.1.5 ± 5.5%, high frequency: 59.4 ± 7.4%, $n$ = 5, $P$ < 0.001). **(f)** Evidence that an NMDA spike rather than a $Ca^{2+}$ spike underlies the supralinearity observed with the high frequency STDP protocol. The supralinearity in response to the STDP protocol is maintained in the presence of voltage-gated $Ca^{2+}$ channel antagonists (nifedipine 5 μM, nickel 50 μM), albeit with reduced magnitude (area under the trace: from 98.6 ± 5.0% in control conditions to 75.8 ± 4.8% in presence of $Ca^{2+}$ channel blockers, $n$ = 4, $P$ < 0.05, paired t test). Blocking NMDARs abolishes the supralinear response. **(g)** The magnitude of the supralinearity was estimated by calculating the ratio between the areas under the traces in the absence and the presence of D-AP5. This analysis reveals that antagonists of voltage-dependent calcium channels lead to a reduction in the responses. Thus, the back propagating APs induced by somatic spiking during the high frequency STDP protocol also activate $Ca^{2+}$ channels, as reported previously[6].

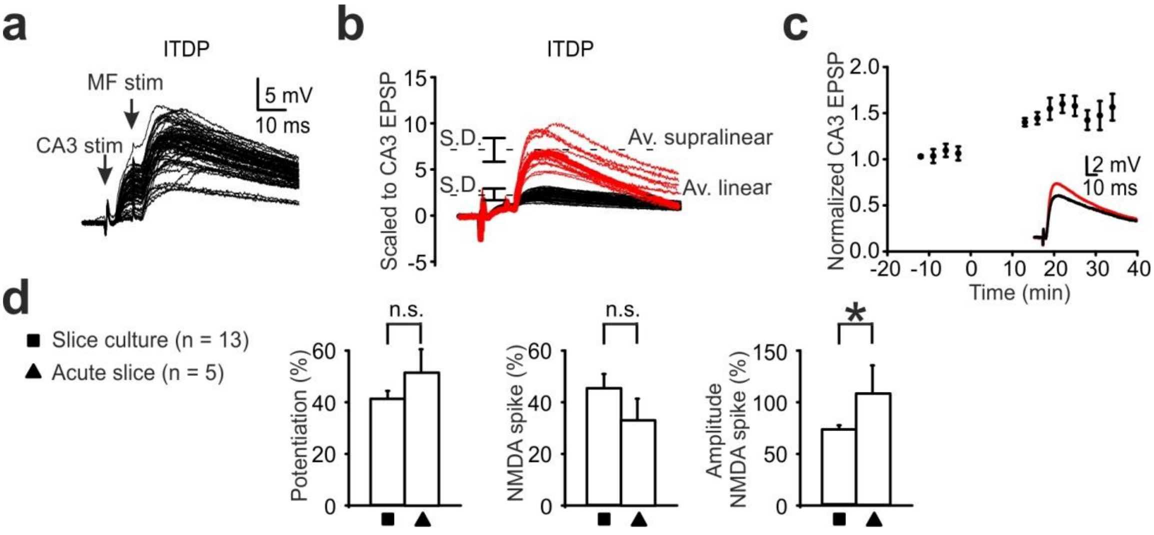


**Supplementary Figure 10**

**NMDA spikes in hippocampal CA3 pyramidal cells exhibit similar properties in acute slices and in organotypic slice cultures.**

**(a)** Responses induced by pairing of a rCA3 EPSP followed by a MF EPSP. **(b)** Scaling to normalize the traces to the amplitude of the rCA3 EPSP reveals linear responses (black) and supralinear responses (red) corresponding to NMDA spikes. **(c)** The presence of NMDA spikes is associated with the induction of LTP Inset: rCA3 EPSPs before (black) and after (red) the ITDP pairing protocol. **(d)** Differences in NMDA spike properties recorded in acute slices (no Fluo-5F) versus cultured slices (Fluo-5F) are not significant except for a slight difference in amplitude. Potentiation: acute slice: 51.8 ± 10.5% after 30 min, *n* = 5/7, slice culture: 41.9 ± 2.3% *n* = 13/16, *P* = 0.08. Percent of supralinear events during 60 rCA3/MF pairings: acute slice: 32.5 ± 8.5%, *n* = 5, slice culture: 45.3 ± 3.2%, *n* = 13, *P* = 0.07. Amplitude of supralinear events normalized to rCA3 EPSP amplitude: acute slice: 105.8 ± 27.3%, *n* = 5, slice culture: 71.5 ± 2.7%, *P* = 0.02.

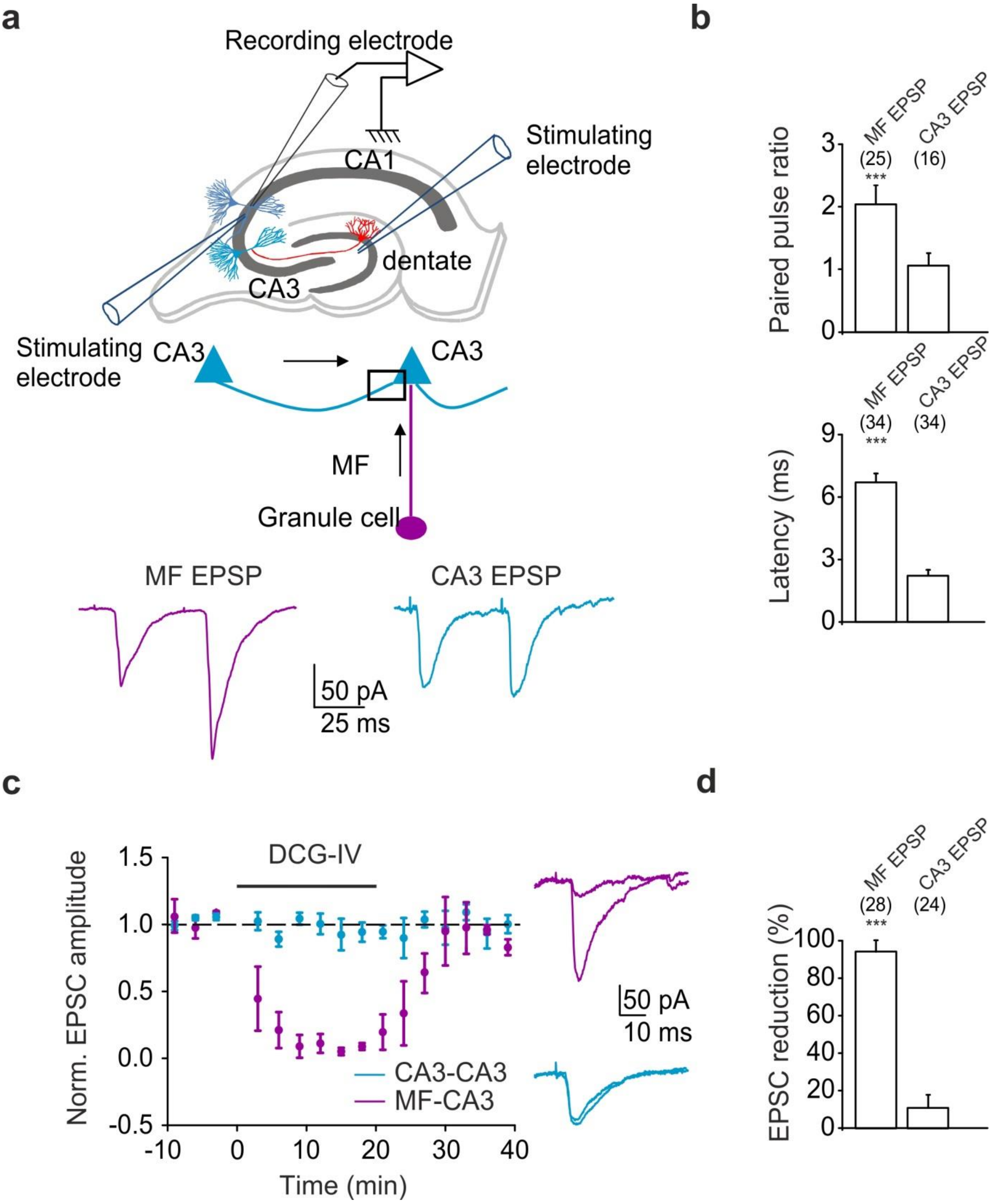


**Supplementary Figure 11**

**Criteria for differentiating between responses evoked by stimulating mossy fibers versus rCA3 fibers.**

**(a)** Experimental recording configuration. Insets show representative EPSCs recorded in a CA3 pyramidal cell that were evoked by paired-pulse stimulation with a 50 ms interval either of mossy fiber or of rCA3 fibers. **(b)** Pooled data. Mossy fiber stimulation results in paired-pulse facilitation (ratio: 2.1 ± 0.8, *n* = 25), whereas rCA3 fiber stimulation induces either paired-pulse facilitation or depression (ratio: 1.2 ± 0.6, *n* = 25, *P* < 0.001, paired t-test).

Moreover, the latency of evoked EPSCs is greater after stimulating mossy fibers as compared to rCA3 fibers (6.7 ± 0.3 ms, versus 2.4 ± 0.1 ms, $n = 34$, $P < 0.001$ paired t-test). **(c)** Bath-application of DCG-IV (2 μM), a Group II metabotropic glutamate receptor agonist that blocks glutamate release from mossy fibers but not from CA3 pyramidal cell terminals, selectively diminishes mossy fiber but not rCA3 responses (MF: 91.3 ± 5.1%, $n = 28$, $P <$ 0.00.1; rCA3: 11.9 ± 6.4%, $n = 24$, $P = 0.2$, paired t-test). **(d)** Pooled data for DCG-IV experiments.
